# Restricted Attentional Capacity within but Not between Sensory Modalities: An Individual Differences Approach

**DOI:** 10.1371/journal.pone.0015280

**Published:** 2010-12-07

**Authors:** Sander Martens, Manasa Kandula, John Duncan

**Affiliations:** 1 Neuroimaging Center, University of Groningen, Groningen, The Netherlands; 2 Department of Neuroscience, University Medical Center Groningen, Groningen, The Netherlands; 3 MRC Cognition and Brain Sciences Unit, Cambridge, United Kingdom; Center for Genomic Regulation, Spain

## Abstract

**Background:**

Most people show a remarkable deficit to report the second of two targets when presented in close temporal succession, reflecting an attentional blink (AB). An aspect of the AB that is often ignored is that there are large individual differences in the magnitude of the effect. Here we exploit these individual differences to address a long-standing question: does attention to a visual target come at a cost for attention to an auditory target (and vice versa)? More specifically, the goal of the current study was to investigate a) whether individuals with a large within-modality AB also show a large cross-modal AB, and b) whether individual differences in AB magnitude within different modalities correlate or are completely separate.

**Methodology/Principal Findings:**

While minimizing differential task difficulty and chances for a task-switch to occur, a significant AB was observed when targets were both presented within the auditory or visual modality, and a positive correlation was found between individual within-modality AB magnitudes. However, neither a cross-modal AB nor a correlation between cross-modal and within-modality AB magnitudes was found.

**Conclusion/Significance:**

The results provide strong evidence that a major source of attentional restriction must lie in modality-specific sensory systems rather than a central amodal system, effectively settling a long-standing debate. Individuals with a large within-modality AB may be especially committed or focused in their processing of the first target, and to some extent that tendency to focus could cross modalities, reflected in the within-modality correlation. However, what they are focusing (resource allocation, blocking of processing) is strictly within-modality as it only affects the second target on within-modality trials. The findings show that individual differences in AB magnitude can provide important information about the modular structure of human cognition.

## Introduction

Limitations on divided attention across different modalities have been the subject of much controversy. While it is well known that information from multiple senses can be integrated very rapidly (e.g., [1]), it remains equivocal whether attention to one modality comes at a cost for a different modality. Whereas there are several early cognitive studies that have shown a cost for cross-modal divided attention [2], [3], [4], [5] there is also considerable evidence demonstrating substantial independence between visual and auditory attentional resources [6], [7], [8], [9], [10], [11], [12], [13], [14], [15], [16].

In this paper we focus on evidence from the attentional blink paradigm, which has proven to be particularly useful in indexing the time-course of attention. In hundreds of experiments it has been shown that when two targets are presented within a rapid stream of non-targets (i.e., distractors), most individuals demonstrate a profound difficulty to report the second target (T2) when presented within 200–500 ms after the first (T1). This interference effect, which is referred to as the attentional blink (AB) in analogy to eye blinks [17], is very robust and can be obtained under a variety of task conditions, using for instance alphanumeric stimuli [18], words [19], pictures [20], and with auditory [16] or tactile stimuli [21]. Consequently, the effect is thought to reflect a very general property of perceptual awareness with broad implications for understanding how the brain perceives a relevant stimulus (for a review, see [22]).

Duncan, Martens, and Ward [16] have shown that the AB occurs in vision as well as in audition when both targets are presented within the same modality. However, when the two targets were presented in different modalities (one in the visual and another in the auditory modality), any temporal restrictions in attentional capacity as reflected in the AB disappeared. Thus, the use of relatively simple, independent visual and auditory stimuli (one-syllable words) that required unspeeded responses, led to an AB within but not between modalities, which strongly suggests the existence of modality-specific limitations rather than an amodal, more central bottleneck.

Although there are a number of studies that have replicated the lack of a cross-modal AB [23], [24], [25], other studies have challenged these findings, reporting significant cross-modal AB effects [26], [27], [28]. A possible explanation for these conflicting results is that in studies finding a cross-modal AB, one of the targets required a speeded response [27], [29], [30] or incorporated a task-switch due to targets differing in task set, target set, target set size, response set, target difficulty, or target-defining features other than modality [26], [27], [28], [31], [32], [33], [34].

Recently, it has been reported that large individual differences exist in AB magnitude [12], [35], [36], [37]. The aim of the current study was to resolve the cross-modal AB controversy described above by taking an individual differences approach combined with the use of equivalent independent targets that differed only in modality.

The primary research question was: if some individuals have large within-modality AB magnitudes, might they also show an AB in the cross-modal case? If cross-modal and within-modal interference arise from the same central amodal bottleneck, individual cross-modal ABs should correlate with individual within-modal AB magnitudes. The lack of such a correlation would suggest that the interference observed in cross-modal conditions is different from that observed in within-modality conditions. A third possibility is that a significant AB is only observed when targets are presented within the same modality, but not when presented in different modalities. Such a finding would provide strong evidence that the AB reflects modality-specific rather than amodal limitations.

The second question that we wanted to address was whether individual differences in AB magnitude within one modality correlate with individual differences in another modality. In other words, does an individual with a large visual AB magnitude also show a large auditory AB magnitude? If not, this would suggest that attentional restrictions within each modality are completely separate.

## Methods

### Participants

Fifty-six volunteers (aged 18–40, mean  = 22.7) recruited from the University of Groningen community participated in the experiment, had Dutch as their native language, normal or corrected-to-normal visual acuity, normal hearing, and no history of neurological problems. The Neuroimaging Center Institutional Review Board approved the experimental protocol and written consent was obtained prior to the experiment. Informed consent was obtained prior to the experiment, and participants received payment of € 11.

### Stimuli and apparatus

The generation of stimuli and the collection of responses were controlled by using E-prime 1.2 software [38] running under Windows XP. Visual and auditory target stimuli consisted of consonant letters excluding “S”, “V”, and “Y”, and distractor stimuli consisted of the digits 0, 2, 3, 4, and 8. Visual stimuli were centrally presented in black (2 cd/m^2^) on a white background (88 cd/m^2^), in one rapid serial visual presentation stream, in uppercase 12-point Times New Roman font on a 19-inch CRT monitor with a 100-Hz refresh rate. The auditory stimuli were the same as those used in Martens et al. [12]. They were digitally recorded and compressed to 120-ms duration, and binaurally presented in a single auditory stream at approximately 83 dB using Sony MDR-V600 headphones.

### Procedure

Each trial began with a message at the bottom of the screen, prompting participants to press the space bar to initiate the trial. When the space bar was pressed the message disappeared immediately and a fixation cross appeared which remained on the screen for 250 ms, followed by two concurrently presented streams, one presented in the visual modality and the other in the auditory modality. Each stream consisted of 16 items.

In each trial, two target letters were randomly presented in any of the two modalities, thus creating four possible target-modality combinations: visual T1 – visual T2 (VV), auditory T1 - auditory T2 (AA), visual T1 – auditory T2 (VA), and auditory T1 – visual T2 (AV). Except for visual targets, the duration of all stimuli was 120 ms. Following Martens et al. [12], we attempted to control task difficulty, keeping mean visual T1 performance equivalent to mean auditory T1 performance, by manipulating the duration of visual targets in the following way. Each block began with a visual target duration of 90 ms, immediately followed by a 30-ms mask (a digit). After the first VV trial, target and mask duration were variable, with target duration ranging from 20 to 100 ms. The sum of target and mask duration was always 120 ms, thereby keeping the interval between the onset of a target and the onset of a subsequent distractor constant. After each within-modality trial a running average of T1 accuracy was calculated. Whenever mean T1 accuracy in the VV condition became 5% higher than the mean T1 accuracy in the AA condition, visual target presentation was decreased by 10 ms and mask duration was increased by 10 ms, thereby making visual target identification more difficult. When mean T1 accuracy in the VV condition became 5% lower than the mean T1 accuracy in the AA modality, visual target presentation duration was increased by 10 ms and masked duration decreased by 10 ms, thereby making visual target identification easier.

The first target was always presented as the fifth item in one of the streams. T2 was the first, second, third, fourth, seventh, or ninth item following T1 (i.e., it was presented at lag 1, 2, 3, 4, 7, or 9, respectively). Thus, the stimulus onset asynchrony (SOA) between the targets randomly varied from 120, 240, 360, 480, 840, to 1020 ms. Each combination of target modality and lag was presented equally often. Target letters were randomly selected with the constraint that T1 and T2 were always different letters. Digit distractors and masks were randomly selected with the constraint that no single digit was presented twice in succession.

After the presentation of the stimulus stream, participants were prompted by a message at the bottom of the screen to type the letters they had seen using the corresponding keys on the computer keyboard. Participants were instructed to take sufficient time in making their responses to ensure that typing errors were not made. Participants were encouraged to type in their responses in the order in which the letters had been presented, but responses were accepted and counted correct in either order.

The experiment started with a block to familiarize participants with each auditory stimulus. All 23 stimuli were presented one by one, in isolation. Participants identified each stimulus by pressing the corresponding key on the keyboard. When all stimuli had been presented once, stimuli that were not correctly identified were presented again in random order, until all stimuli were identified correctly. Subsequently, 4 practice blocks were presented, consisting of 12 trials each. The first practice block only contained AA trials, the second block only VV trials, the third block only VA and AV trials, and the fourth block contained all types of trials. After these practice blocks, six testing blocks were presented, consisting of 120 trials each, resulting in 30 repetitions of each lag-modality combination. After each block, participants were allowed to take a short break. The experiment took approximately 90 minutes to complete.

## Results and Discussion

When appropriate, Greenhouse-Geisser-corrected *p* values are reported. Eight participants were rejected from analysis due to high error rates in identifying T1 and T2.

**Figure 1:**
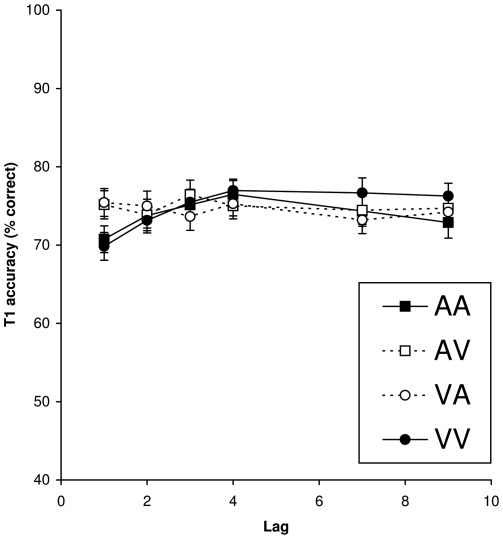
T1 accuracy. Mean percentage correct report of T1 as a function of lag when T2 was presented in the same (solid lines) or in a different (dotted lines) sensory modality. Error bars reflect standard error of the mean.

Figure 1 shows T1 identification performance (dotted lines) as a function of the interval between the two targets (lag) within the visual and auditory modalities. Overall mean T1 performance was 74.6%. A repeated measures analysis of variance (ANOVA) of T1 performance with condition (VV, AA, VA, AV) and lag (1, 2, 3, 4, 7, 9) as a within-subjects factor revealed a significant effect of lag, *F*(5, 200) = 3.39, *MSE*  = 50.55, *p*<.01, *η^2^_p_* = .08, such that performance was lower at lag 1 than at the other lags. No significant main effect of condition (*p* = .24) was found, but the Condition × Lag interaction was borderline significant, *F*(15, 600) = 1.83, *MSE*  = 58.22, *p* = .053, *η^2^_p_* = .04, such that performance at lag 1 was decreased in the within-modality conditions, but not in the between-modality conditions. This suggests that there was more direct competition between two successive targets when presented within the same modality than between modalities.

**Figure 2:**
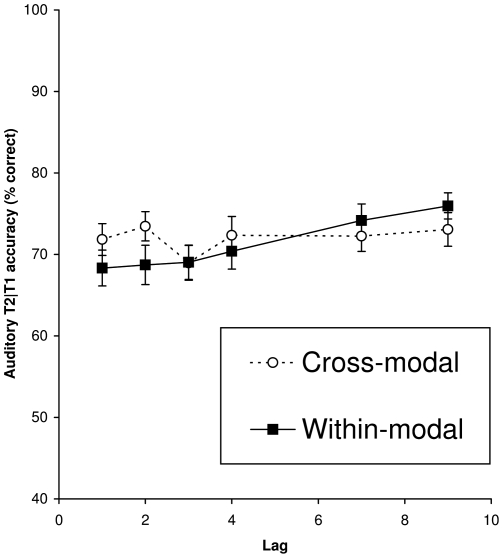
Auditory T2 accuracy. Mean percentage correct report of an auditory T2 given correct report of T1 as a function of lag when presented within (solid line) or between modalities (dotted line).

**Figure 3:**
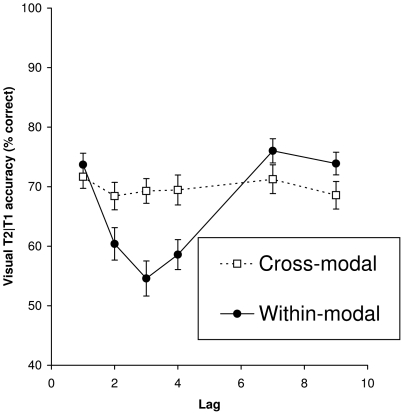
Visual T2 accuracy. Mean percentage correct report of a visual T2 given correct report of T1 as a function of lag when presented within (solid line) or between modalities (dotted line).

As can be seen in Figures 2 and 3, an AB occurred when targets were presented within either the auditory (Figure 2, solid line) or the visual (Figure 3, solid line) modality. In contrast, there was a lack of an AB when targets were presented in different modalities (dotted lines). An ANOVA on T2 performance given correct T1 report, with condition and lag as within-subjects factors, revealed significant main effects of condition, *F*(3, 5) = 7.33, *MSE*  = 267.00, *p*<.001, *η^2^_p_* = .16; lag, *F*(5, 200) = 19.20, *MSE*  = 108.35, *p*<.001, *η^2^_p_* = .32; and a significant Condition × Lag interaction, *F*(15, 600) = 10.69, *MSE*  = 83.52, *p*<.001, *η^2^_p_* = .21, reflecting differences in performance when targets were presented in the same or in different sensory modalities.

An ANOVA on within-modality T2 performance showed significant main effects of condition, *F*(1, 40) = 11.38, *MSE*  = 299.90, *p* = .002, *η^2^_p_* = .22; and lag, *F*(5, 200) = 27.96, *MSE*  = 116.32, *p*<.001, *η^2^_p_* = .41; and a significant Condition × Lag interaction, *F*(5, 200) = 14.95, *MSE*  = 86.28, *p*<.001, *η^2^_p_* = .27, reflecting the occurrence of an AB that was larger in the visual modality than in the auditory modality. A separate ANOVA on T2 performance in the auditory within-modality (AA) condition, revealed a significant main effect of lag, *F*(5, 235) = 4.43, *MSE*  = 110.72, *p* = .001, *η^2^_p_* = .09, confirming the presence of an AB within the auditory modality.

An ANOVA on between-modality T2 performance only revealed a significant effect of condition, *F*(1, 40) = 5.10, *MSE*  = 275.50, *p* = .03, *η^2^_p_* = .11, such that overall performance of auditory T2s was slightly better (72.8%) than that of visual T2s (69.4%). Importantly though, neither a significant main effect of lag (*p* = .24), nor a significant Condition × Lag interaction was observed (*p* = .24).

Intra-individual stability of performance was checked on odd and even number trials for all participants. For T1, the Spearman-Brown prophecy coefficients were .88, .94, .90, and .90 for the AA, VV, AV, and VA condition, respectively. For T2|T1, Spearman-Brown prophecy coefficients were .80, .90, .91, and .88 for the AA, VV, AV, and VA condition, respectively. These values reflect stable within-subject performance, similar to that observed in previous studies [12], [35], [37].

**Figure 4:**
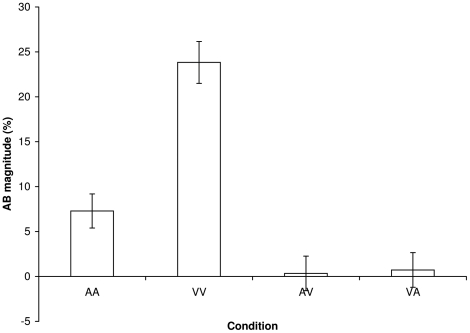
AB magnitude. AB magnitudes within (AA and VV) and between modalities (AV and VA).

For each individual and condition, AB magnitude was computed according to the following formula:
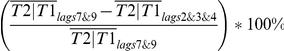



That is, the percentage of decrement in T2 performance within the AB period (lags 2, 3, and 4) relative to that outside the AB period (lags 7 and 9) was calculated, and the resulting AB magnitudes are shown in Figure 4. One-sample t-tests revealed that AB magnitude was significantly different from zero in both within-modality conditions (*p*s <.001), but not in the between-modalities conditions (*p*s >.71). When only the 25% of participants with the largest within-modality ABs (mean of AB magnitude in AA and VV conditions) were selected, mean AB magnitudes were 21.5% in the AA, 40.1% in the VV, 3.4% in the AV, and 4.8% in the VA condition, respectively. Again, one-sample t-tests revealed that AB magnitude was significantly different from zero in both within-modality conditions (*p*s <.001), but not in the between-modalities conditions (*p*s >.20).

Pearson product-moment correlations were computed and revealed a significant positive correlation between individual AB magnitudes within each modality, *r* = .37, *p*<.01 (two-tailed), such that participants with a relatively large visual AB also tended to be show a large auditory AB. This may seem to suggest the existence of a common amodal pool of resources. However no significant relation was found when AB magnitude within modalities was correlated with AB magnitude between modalities, *r* = .18, *p* = .23, providing strong evidence against an amodal limited-capacity bottleneck as the underlying cause of the AB. The Spearman-Brown prophecy coefficients were .57 and .28 for AB magnitude within- and between sensory modalities, respectively. The relatively low intra-individual stability in cross-modal AB magnitude suggests that the variability in cross-modal AB magnitude merely reflects random noise. In other words, under the current experimental conditions there is no evidence for a cross-modal AB.

### General Discussion

An aspect of the AB that is often ignored is that there are large individual differences in the magnitude of the effect (e.g., [36]). In the current study, we exploited these individual differences to address a long-standing question: does attention to a visual target come at a cost for attention to an auditory target (and vice versa)? More specifically, the goal of the current study was to investigate a) whether individuals with a large within-modality AB also show a large cross-modal AB, and b) whether individual differences in AB magnitude within different modalities correlate or are completely separate.

While minimizing differential task difficulty and chances for a task-switch to occur between the targets, using a randomized within-subjects design we observed a significant AB effect when targets were both presented within the auditory or visual modality. A positive correlation was found between an individual's auditory and visual AB magnitude, and at first sight, this may seem to suggest a common amodal source of interference.

Importantly, however, when the two targets were presented in different modalities, no interference that was time-locked to the presentation of the targets occurred, reflecting the absence of a cross-modal AB effect. The commonly observed decreased T1 performance at lag 1 was found in within-modality, but not between-modality conditions, indicating modality-specific interference between the two targets. Moreover, individual cross-modal AB magnitude did not correlate with individual within-modal AB magnitude. Even the 25% of participants with the largest within-modality ABs did not show a significant cross-modal AB effect. Finally, the relatively low intra-individual stability of cross-modal AB magnitude on odd and even trials suggests that the observed cross-modal variability in AB magnitude between individuals probably reflected random noise. Taken together, the results suggest that under the current experimental conditions, a major source of attentional restriction must lie in modality-specific sensory systems.

These findings replicate and extend previous reports of an AB within- but not between visual and auditory modalities [16], [23], [24]. Whereas for instance the original study by Duncan and colleagues [16] used different target sets, different target locations, a varying number of stimulus streams, and different groups of participants for each condition, the current study addressed these potential methodological issues by employing a within-subjects design, incorporating both within- and between-modality conditions within a single experiment, and randomly mixed all conditions across trials (rather than blocks of trials). That is, even after participants had received the first target on a given trial, the modality of the upcoming T2 (visual or auditory) remained unpredictable. In addition, none of the targets required a speeded response [27], [29], [30]. Whereas previous findings of time-locked cross-modal interference may have been caused by some sort of task-switch [26], [27], [28], [31], [32], [33], [34], chances for a task-switch to occur were minimal in the current study as there was no change in task set, target set, target set size, response set, target difficulty, or any target-defining feature other than modality. To the best of our knowledge, this is the first study taking an individual differences approach to resolve the cross-modal AB controversy, revealing modality-specific restrictions in temporal attention within but not between sensory modalities in a within-subjects design, while controlling for the above-mentioned confounds.

In a previous study on individual differences in AB magnitude, we found that visual non-blinkers do show an auditory AB, suggesting restrictions within the visual and the auditory modality to be independent [12]. Indeed, the significant but relatively modest correlation between individual within-modality ‘blinks’ suggest that strong auditory blinkers are not always strong visual blinkers (and vice versa). Nevertheless, this modest correspondence in within-modality AB magnitudes, together with the lack of a cross-modal AB, suggest that in most (but apparently, not all) individuals there is a common delay in the *modality-specific* re-allocation of attention for T2, or alternatively, a similar protection process that inhibits *modality-specific* sensory input.

It has also been suggested that strong blinkers may be especially committed or focused in their processing of T1 [39], [40], [41], and to some extent that tendency to focus can apparently cross modalities. Importantly though, whatever they are focusing (resource allocation, blocking of processing, etc.) is strictly within-modality. Since participants did not know which T2 will be presented at the moment of receiving T1, it seems plausible to assume that, for strong blinkers, the same focused T1 processing occurs on both types of trials (within-modality and cross-modality) - but it only affects T2 on within-modality trials. The current study shows that individual differences in AB magnitude can provide important information about the modular structure of human cognition.
